# Latitudinal Variation in the Molecular Diversity and Community Composition of Symbiodiniaceae in Coral From the South China Sea

**DOI:** 10.3389/fmicb.2019.01278

**Published:** 2019-06-18

**Authors:** Biao Chen, Kefu Yu, Jiayuan Liang, Wen Huang, Guanghua Wang, Hongfei Su, Zhenjun Qin, Xueyong Huang, Ziliang Pan, Wenwen Luo, Yanqiu Luo, Yinghui Wang

**Affiliations:** ^1^Guangxi Laboratory on the Study of Coral Reefs in the South China Sea, Guangxi University, Nanning, China; ^2^Coral Reef Research Center of China, Guangxi University, Nanning, China; ^3^School of Marine Sciences, Guangxi University, Nanning, China

**Keywords:** coral reefs, Symbiodiniaceae, ITS2-rDNA, next generation sequencing, molecular diversity, biogeographical partten, South China Sea

## Abstract

Coral reefs are continuing to decline worldwide due to anthropogenic climate change. The study of the molecular diversity and biogeographical patterns of Symbiodiniaceae, is essential to understand the adaptive potential and resilience of coral–algal symbiosis. Next generation sequencing was used to analyze the Symbiodiniaceae rDNA internal transcribed spacer 2 marker genes from 178 reef-building coral samples in eight coral habitats across approximately 13° of latitude in the South China Sea (SCS). A total of three Symbiodiniaceae genera, *Cladocopium*, *Durusdinium*, and *Gerakladium*, as well as 31 dominant Symbiodiniaceae types, were identified. Symbiodiniaceae richness, diversity, and community composition varied according to latitude; intermediate and low latitude coral reefs (IR and LR) have higher Symbiodiniaceae richness and diversity than high latitude coral habitats (HC and HR). A PERMANOVA analysis found significant differences in the Symbiodiniaceae community composition in the SCS (*F* = 14.75, *R*^2^ = 0.20, *p* = 0.001 < 0.01). The major dominant Symbiodiniaceae types were C1 in the HC and the HR, C1/Cspc/C50/C15 and D1 in the IR, and C3u and C15 in the LR. Canonical correspondence analysis showed that the relative abundance of different Symbiodiniaceae types is affected by multiple environmental factors. Phylogenetic analysis indicated that the Symbiodiniaceae type *Cladocopium*, which shared common ancestors, shows similar environmental adaptability. Based on these results, we suggest that coral host species played a relatively small role in the identity of the dominant Symbiodiniaceae type. Therefore, the biogeographical patterns of Symbiodiniaceae may be mainly affected by environmental factors. Our research provides a comprehensive overview of the biogeography of Symbiodiniaceae in the SCS, where coral communities and reefs are widely distributed across different latitude regions and have variable environmental conditions. Our data will provide support for further study of the regional diversification of Symbiodiniaceae and the ecological resilience of the coral-Symbiodiniaceae symbioses.

## Introduction

Coral reefs are the most diverse ecosystems in the oceans, and they provide habitats for at least 30% of marine organisms (Reakakudla et al., 1997). They are also the most diverse symbiotic ecosystems on earth (Blackall et al., 2015). The ability of reef-building corals to build reef structures depends on Symbiodiniaceae, which provides up to 95% of host respiratory demand (Falkowski et al., 1984; Muscatine et al., 1984). However, this special symbiotic relationship is threatened by anthropogenic climate change (Carpenter et al., 2008; Hughes et al., 2017; Langlais et al., 2017). When the external environment changes drastically, the coral suffers the loss of symbionts, and coral bleaching occurs (Glynn, 1993; Rowan et al., 1997; Hoegh-Guldberg, 1999; Baker, 2003; Hughes et al., 2003, 2017; Langlais et al., 2017). As anthropogenic climate change continues to increase, coral reef ecosystems are being seriously threatened (Hoegh-Guldberg et al., 2007). The strongest El Niño event recorded occurred in 1997–1998, and the highest global sea surface temperature (SST) that was experienced in 1998 directly led to global-scale coral bleaching, with an effective loss of 16% in coral coverage (Wilkinson, 2002). Moreover, damage to coral reefs by anthropogenic climate change is cyclical, with increased frequency and longer-lasting effects. The two global bleaching events that occurred in 2010 and 2015 have once again seriously affected global coral reef ecosystem (Hughes et al., 2017). The bleaching event in 2015 lasted for 2 years and became the longest-lasting global bleaching event in history (Eakin et al., 2016; Hughes et al., 2017; Langlais et al., 2017). According to the statistics, the collapse of coral–algal symbiosis since the 1970s has been due to climate change, which caused the coral cover to dramatically decline by approximately 50–80% worldwide (Gardner et al., 2003; Bruno and Selig, 2007; Silverstein et al., 2015). In addition to the increase in SST, the health of coral reef ecosystems is also threatened by a number of other factors, including ocean acidification and eutrophication (Goiran et al., 1996; Lapointe, 1997). However, different kinds of Symbiodiniaceae have unique eco-physiological and environmental adaptability characteristics that result in differences in the tolerances of their symbiosis systems (LaJeunesse et al., 2010, 2018; Hume et al., 2016). Therefore, Symbiodiniaceae are ecologically diverse and have established symbiotic relationships with different coral hosts that exhibit distinct environmental tolerances at large geographical scales (Cantin et al., 2009; LaJeunesse et al., 2010; Baker et al., 2013; Ziegler et al., 2015). Moreover, there is some evidence supporting the “adaptive bleaching hypothesis,” which suggests that corals may be able to acclimate to future climate change by shuffling or switching their symbionts (Baker, 2004; Fautin and Buddemeier, 2004; Stat et al., 2006). However, the shuffling and switching of Symbiodiniaceae community composition does not seem to have saved more corals in the recent global bleaching event (Perry and Morgan, 2017; Hughes et al., 2018). This results from the fact that coral-Symbiodiniaceae symbioses are stable (Thornhill et al., 2006; Hume et al., 2015; Ziegler et al., 2017), which, however, does not eliminate the possibility of symbiont community composition change driven by SST and light stress (Silverstein et al., 2015; Boulotte et al., 2016). For example, *Orbicella faveolata*, an endangered species from areas of the Caribbean Sea and Gulf of Mexico such as the Florida Keys (Florida, United States) (Manzello et al., 2018), retained some of the coral species that are vital for the heavily degraded coral reef ecosystem.

In the past, the Symbiodiniaceae family (formerly the Symbiodinium genus) was divided into nine clades (A–I) by a molecular taxonomy study (Pochon and Gates, 2010). A recent study proposed that evolutionarily divergent zooxanthellae “clades” are equivalent to genera in the family Symbiodiniaceae, and consequently seven of these were formally described as *Symbiodinium* (formerly Clade A); *Breviolum* (formerly Clade B); *Cladocopium* (formerly Clade C); *Durusdinium* (formerly Clade D); *Effrenium* (formerly Clade E); *Fugacium* (formerly Clade F); and *Gerakladium* (formerly Clade G) (on the basis of genetics and ecology, clades H and I were not formally described as genera; LaJeunesse et al., 2018). Of these, *Symbiodinium*, *Breviolum*, *Cladocopium*, and *Durusdinium* generally form symbiotic relationships with corals (Pochon et al., 2004; Ziegler et al., 2017). These genera can be further divided into subclades or types with distinct genetic, physiological, and ecological differences using high-resolution molecular markers (LaJeunesse et al., 2014, 2018) such as rDNA internal transcribed spacer regions (ITS1 and ITS2) (LaJeunesse and Trench, 2000; LaJeunesse, 2001; Oppen, 2001; Arif et al., 2014), the hypervariable regions of domain V of the chloroplast large subunit (cp23S) (Santos et al., 2002; Santos and Coffroth, 2003; Pochon et al., 2006), and the chloroplast psbA non-coding region (psbA^ncr^) (LaJeunesse and Thornhill, 2011; Reimer et al., 2017). Of these markers, the ITS2 region remains the most generally used marker for the determination of Symbiodiniaceae diversity. However, many challenges remain in the application of ITS2, including intragenomic variation (IGV), pseudogenes, and PCR artifacts that can interfere with diversity assessments and lead to overestimations of a magnitude of approximately six to eight times (Apprill and Gates, 2007; Thornhill et al., 2007; Stat et al., 2011). Therefore, it is important to avoid the interference caused by these factors, especially the interference caused by IGV (Thornhill et al., 2007; Sampayo et al., 2010; Ziegler et al., 2017). Although PCR-DGGE can be used to identify dominant ITS2 variants, which in many cases represent Symbiodiniaceae types, or even species (LaJeunesse, 2001; Arif et al., 2014; Ziegler et al., 2017; Thornhill et al., 2017), its use is limited by its sensitivity, which can only identify symbionts with a relative abundance of > 5–10% (Thornhill et al., 2006). Recently, next generation sequencing (NGS) has been applied to assess Symbiodiniaceae ITS2 diversity, which has greatly improved the resolution of different ITS2 to < 5%, or potentially to 1%) (Arif et al., 2014; Thomas et al., 2014; Tong et al., 2017; Ziegler et al., 2017). However, the ITS2 is a multicopy marker because of the tandem repeat arrangement of rRNA genes; consequently, the identification of IGV remains an issue (Thornhill et al., 2007; Sampayo et al., 2010). Arif et al. (2014) established a data processing method for Symbiodiniaceae ITS2 NGS data and suggested 97% to be the optimal cut-off value for operational taxonomic unit (OTU) clusters. This improved the objectivity, comparability, and simplicity of Symbiodiniaceae diversity assessments. In addition, many studies have only considered ITS2 variants with > 5% abundance in at least one sample for diversity analysis, which allows for comparison with the results of DGGE from previous studies, and can reduce IGV interference (Tong et al., 2017; Zhou et al., 2017; Ziegler et al., 2017; Cai et al., 2018). At present, NGS has been used to evaluate the diversity and community composition of Symbiodiniaceae in coral habitats around the world, and to reveal their biogeographic and ecological patterns in different regions (Thomas et al., 2014; Klepac et al., 2015; Stat et al., 2015; Ziegler et al., 2017).

**Figure 1:**
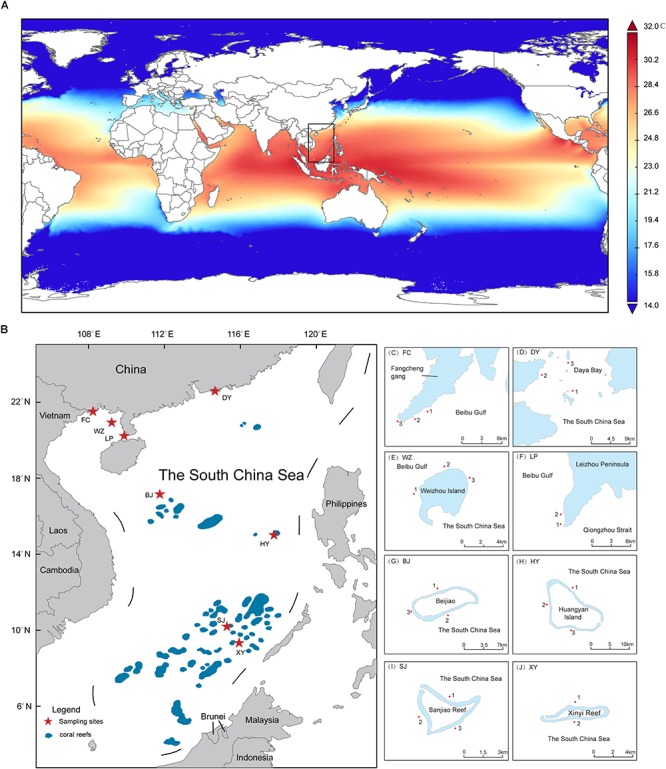
The range of coral reef distribution and sampling areas in the South China Sea (SCS). **(A)** Annual average SST (2008–2016) in the SCS indicate that which have difference among distinct latitude areas **(B)** Map of the whole SCS with labeled coral reef area. The sampling sites is marked by star. **(C–J)** The details of sampling sites in the SCS. The map was constructed using software ArcGIS (ver. 10.1).

Coral reefs in the SCS have high biological diversity. The SCS is located on the northern edge of the “Coral Triangle,” which borders the high latitude coral reefs of Japan in the north and the coral diversity center of Indonesia in the south (Spalding et al., 2001) (Figure 1A,B). Coral reefs are widely distributed through the SCS, from the Zengmu Reef (∼4°N) near the equator, to the Leizhou Peninsula and Weizhou Island (∼20–21°N) in the northern part of the SCS (Wang and Li, 2009; Yu, 2012). Higher latitudes such as Hong Kong, Daya Bay, Dongshan, and Fangchenggang (∼21–23°N) have also been found to have non-reefal coral communities (Chen et al., 2007; Dong et al., 2008; Ng and Ang, 2016). However, the results of ecological monitoring show that the coral reefs in the SCS have degraded rapidly over the past few decades, following the same trend in degradation of coral reefs that has been seen globally (Gardner et al., 2003; Bellwood et al., 2004; Yu, 2012; Zhao et al., 2013, 2016). Therefore, it is essential to evaluate both the potential adaptability of the coral–algal symbiotic system and the factors that affect this in the SCS. Molecular research on Symbiodiniaceae can be used to evaluate the diversity and biogeographical patterns of this family (Baker, 2004; Rowan, 2004; Tong et al., 2017; Cai et al., 2018). In the past, molecular studies of Symbiodiniaceae have focused on coral–algal symbiosis in Hong Kong, Sanya, and southern Taiwan, which are all in the northern SCS, and have shown low diversity in these areas (Chen et al., 2005; Zhou and Huang, 2011; Ng and Ang, 2016). The coral growth areas in the northern SCS are close to the Chinese mainland and are therefore affected by various factors such as seasonal low temperatures and lower salinity that may cause Symbiodiniaceae diversity or community composition to be obviously different from those in coral reef areas further from the Chinese mainland. In the case of intermediate (Xisha Islands and Zhongsha Islands) and low (Nansha Islands) latitude areas in the SCS, studies have indicated that coral–Symbiodiniaceae symbiosis is shaped by high temperatures in these areas, and the relative abundance of *Durusdinium* (Clade D) increased in *Montipora* and *Galaxea* corals (Tong et al., 2017). Moreover, there is a significant difference in Symbiodiniaceae community composition between high latitudes and intermediate or low latitudes (Huang et al., 2006, 2011; Dong et al., 2009). In addition, these studies used traditional restricted fragment length polymorphisms and DGGE (Chen et al., 2005; Huang et al., 2006, 2011; Dong et al., 2009; Zhou and Huang, 2011; Ng and Ang, 2016), while NGS has not been widely used, so that the evaluation of the diversity and community composition of Symbiodiniaceae lacks sensitivity (Arif et al., 2014; Edmunds et al., 2014; Thomas et al., 2014; Boulotte et al., 2016). As coral reefs in the SCS are distributed across a large spatial scale, the coral–Symbiodiniaceae symbiosis at different latitudes is affected by different environmental factors, such as SST (Hoegh-Guldberg, 1999), light intensity (Rowan et al., 1997; Hoegh-Guldberg, 1999), nutrition concentration (Hoegh-Guldberg, 1999; Gustafsson et al., 2015), and salinity (Veron, 1986; Hoegh-Guldberg, 1999; Gustafsson et al., 2015). Because the coral reefs are distributed across a large spatial scale, our understanding of the molecular diversity and biogeographical patterns of Symbiodiniaceae in the SCS is limited.

In this study, the sampling areas selected included eight coral habitats (six coral reefs and two coral communities) in the SCS, spanning 13° of latitude (Figure 1). NGS was used to analyze the diversity and community composition of Symbiodiniaceae. This study attempted to gain a more complete understanding of the diversity, community composition, phylogenetic relationships, and environmental impact factors at different latitudes in the SCS. It also attempted to understand the molecular diversity and biogeographical patterns of Symbiodiniaceae, as well the factors that impact them. This study may provide vital information that will assist future research into the adaptability and resilience of the coral-Symbiodiniaceae symbiosis to respond to climate change.

## Materials and Methods

Clustering analysis of SST (°C) data from 2008 to 2016 collected by the NASA Giovanni satellite^1^ shows that the sampling areas can be divided into three latitude regions (Supplementary Table 3). Clustering analysis was performed using the Ward method, with a squared Euclidean distance as a metric. Furthermore, depending on whether or not the corals build reefs in high latitude regions, they were further divided into high latitude coral communities (non-reefal) and high latitude coral reefs. Therefore, the sampling areas was finally divided into four latitude regions (Table 1).

**Table 1:** Coral samples from the South China Sea (SCS) including sampling regions, climate, coral habitats, coordinates, environmental factors (contain SST, Chl *a*, PAR, SAL, and KD), coral samples information and collected depth.

Regions	Climate	sampling coral habitats	Coordinates	Environmental factors					Coral species	The number of samples	sampling dates	Depth
								
				SST/ °C (SD)	Chl *a*/ mg⋅m^-3^ (SD)	PAR/ E⋅m^-2^⋅s^-1^ (SD)	SAL/ ‰ (SD)	KD/ m^-1^ (SD)				
High latitude coral communities (HC)	Subtropical	Daya Bay (DY)	E114°33′–114°39′, N22°34′–22°39′	24.4 (4.4)	2.105 (0.65)	37.126 (8.44)	32.6 (0.14)	0.159 (0.05)	*P. lutea*	6	2015.08	2–15 m


									*F. palauensis*	5		
									*P. versipora*	4		
									*M. efflorescens*	5		
									*A. formosa*	5		
		Fangchenggang (FC)	E108°31′_108°33′, N21°56′ _21°58′	24 (4.9)	1.688 (0.68)	35.952 (9.31)	30 (0.15)	0.143 (0.05)	*F. palauensis*	5	2016.08	4–12 m


									*P. versipora*	6		
High latitude coral reefs (HR)		Weizhou Island (WZ)	E109°04′_109°08′, N21°00′ _21°04′	25.5 (4.4)	2.235 (0.55)	37.561 (8.61)	32.4 (0.03)	0.174 (0.04)	*P. lutea*	7	2015.08	2–15 m


									*F. palauensis*	6		
									*P. versipora*	3		
									*M. efflorescens*	6		
									*A. formosa*	6		
		Leizhou Peninsula (LP)	E109°54′–109°55′, N20°13′–20°18′	25.8 (4.1)	2.75 (0.27)	38.58 (9.02)	32.8 (0.05)	0.201 (0.02)	*P. lutea*	3	2015.08	2–12 m


									*P. versipora*	2		
									*M. efflorescens*	1		
Intermediate latitude coral reefs (IR)	Tropical	Beijiao (BJ)	E111°28′–111°31′, N17°06′–17°07′	27.6 (3.8)	0.156 (0.09)	42.553 (7.86)	33.3 (0.05)	0.02 (0.01)	*P. lutea*	6	2015.06	5–15 m


									*F. palauensis*	4		
									*P. versipora*	4		
									*M. efflorescens*	4		
									*A. formosa*	4		
Low latitude coral reefs (LR)		Huangyan Island (HY)	E117°44′–117°50′, N15°06′–15°13′	28.8 (1.2)	0.359 (0.42)	43.657 (5.45)	34.1 (0.04)	<0.01 (0.04)	*P. lutea*	7	2015.05	5–15 m


									*F. palauensis*	5		
									*P. versipora*	6		
									*M. efflorescens*	3		
									*A. formosa*	6		
		Sanjiao Reef (SJ)	E115°16′–115°19′, N10°10′–10°13′	29 (0.9)	0.147 (0.11)	42.654 (5.51)	33.3 (0.03)	<0.01 (0.01)	*P. lutea*	5	2016.05	5–15 m


									*F. palauensis*	5		
									*P. versipora*	6		
									*M. efflorescens*	7		
									*A. formosa*	7		
		Xinyi reef (XY)	E115°54′–115°58′, N9°20′–9°21′	29.1 (0.9)	0.124 (0.05)	43.93 (4.95)	33.1 (0.02)	<0.01 (0.01)	*P. lutea*	4	2016.05	5–15 m


									*F. palauensis*	6		
									*P. versipora*	6		
									*M. efflorescens*	6		
									*A. formosa*	7		

Other environmental data including aqua-MODIS chlorophyll *a* concentration (Chl *a*, mg⋅m^-3^), photosynthetically active radiation (PAR, E⋅m^-2^⋅s^-1^), and the diffuse attenuation coefficient for downwelling irradiance at 490 nm (KD, 1/m) from 2008 to 2016 were also acquired from the NASA Giovanni satellite (Table 1 and Supplementary Table 3). In addition, salinity (SAL, ‰) was measured *in situ* using an ES-421 conductance salinity meter (Atago, Tokyo, Japan) (Table 1 and Supplementary Table 3). These environmental datasets were used in the canonical correlation analysis (CCA).

### High Latitude Coral Communities

Fangchenggang (FC, Figure 1C and Table 1) is located in the northern part of Beibu Gulf from the SCS and near Vietnam. We first discovered non-reefal coral community distribution in this area. Daya Bay (DY, Figure 1D and Table 1) also have not developed into extensive coral reefs and the coral cover has declined significantly between 1983 (76.6%) and 2008 (15.3%) (Chen et al., 2009). The low winter SST is the possible restrictive factor that has prevented greater development of the DY coral communities (Chen et al., 2009). FC and DY were classified as high latitude coral communities (HC), since coral communities are sporadic and corals can not build reefs, affected by the seasonal low temperature.

### High Latitude Coral Reefs

Weizhou Island (WZ, Figure 1E and Table 1) and Leizhou Peninsula (LP, Figure 1F and Table 1) have coral communities and developed into extensive coral reefs. The average live coral cover declined from 50 to 6.02% (1984–2015) and the rate of decline was 1.42%⋅y^-1^ (Wang, 2017). LP is about 110 km southeast of WZ and the coral reefs of LP displayed patchy distribution and live coral coral cover change uneven. According to the latest data, the average living coral cover was only 12.1% in LP, which varies from 0 to 38%. Therefore, the distribution of coral was uneven in LP (Huang et al., 2011). WZ and LP are located at the northern margin of the SCS and are partly affected by seasonal low temperature. So these two areas were classified as high latitude coral reefs (HR).

### Intermediate Latitude Coral Reefs

Beijiao (BJ, Figure 1G and Table 1) is located in the northern part of the Xisha Islands. About coral cover, it has declined obviously. The average live coral cover of Xisha Islands declined from 65 to 7.93% (2005–2009) (Wu et al., 2011). According to the data of Giovanni satellite, the monthly average SST of BJ was 27.6°C, so it was classified as intermediate latitude coral reefs (IR).

### Low Latitude Coral Reefs

Huangyan Island (HY, Figure 1H and Table 1), Sanjiao Reef (SJ, Figure 1I and Table 1) and Xinyi Reef (XY, Figure 1J and Table 1) were classified as low latitude latitude coral reefs with relatively high heat stress. The live coral cover of HY was low, which was only 9.54% in the reef slope and 0.24% in the lagoons (2015). In addition, before this study, there was not coral reef ecological survey conducted in XY and SJ, so we did not collect the data of live coral cover. According to the results of clustering analysis of SST, HY, SJ, and XY were classified as low latitude coral reefs (LR).

### Sample Collection

A total of 178 coral samples from five species were collected from a depth range of 2–15 m using SCUBA. Hammers and chisels were used to collect coral fragments. The collected samples were coral species that are widely distributed throughout the SCS, including *Porites lutea* (*n* = 38), *Favia palauensis* (*n* = 36), *Plesiastrea versipora* (*n* = 37), *Montipora efflorescens* (*n* = 32), and *Acropora formosa* (*n* = 35) (Table 1 and Supplementary Table 1). The collected coral fragments were transferred to cryotubes after being rinsed with sterile seawater (35‰ sterile seawater was used to clean the surface of coral samples, to prevent contamination by free-living Symbiodiniaceae found in seawater) and DMSO/NaCl Buffer was added at a ratio of 4:1 buffer to tissue ratio (Gaither et al., 2011). After the above operation, the cryotubes containing the samples were stored in a 4°C refrigerator prior to DNA extraction.

### DNA Extraction and PCR Amplification

In the pretreatment stage, the coral fragments were ground in a liquid nitrogen environment with a mortar and pestle, and the homogenate and tissue were pelletized by centrifugation at 12,000 *g*. A DNeasy Plant Mini Kit (QIAGEN, Hilden, Germany) was used to extracted DNA from pellets, following the manufacturer’s instructions. The DNA obtained was used as a template for PCA after filtering for quality and purity. ITSintfor2 (5′ GATTGCAGA ACTCCGTG-3′) (LaJeunesse and Trench, 2000) and ITS2-reverse (5′ GGGATCCATA TGCTTAAGTT CAGCGGGT-3′) (Coleman et al., 2010) were used as primers to conducted PCR amplification of the ITS2 region of the Symbiodiniaceae rDNA. An ABI GeneAmp 9700 thermal cycler (Thermo Fisher Scientific, Waltham, MA, United States) was used as a PCR reaction system, and the reactions were conducted under the following conditions: 3 min at 95°C, followed by 35 cycles of 95°C for 30 s, 55°C for 30 s, 72°C for 45 s, and a final extension at 72°C for 10 min. PCRs were run in triplicate per sample, which were conducted using a 20 μL reaction volume of TransGen AP221-02 (TransGen Biotech, Beijing, China) containing: 4 μL 5 × FastPfu Buffer (TransGen Biotech, Beijing, China), 2.5 mM dNTPs, 0.8 μL (5 μm) forward primer, 0.8 μL (5 μm) reverse primer, 0.4 μL FastPfu DNA Polymerase (TransGen Biotech, Beijing, China), and 10 ng template DNA; the final volume was adjusted to 20 μL using ddH_2_O. PCR products stained with loading buffer were run on a 2% agarose gel. Purified amplicons were combined in equimolar amounts, and a paired-end sequenced (PE)300bp × 2 strategy was used on an Illumina MiSeq platform (Illumina, San Diego, CA, United States); analyses were undertaken at Majorbio Biopharm Technology Co., Ltd., Shanghai, China. All sequences were submitted into the NCBI Sequence Read Archive (SRA) database (Accession number: SRP162001 and SRP 181784).

### Data Processing and Bioinformatics Analysis

Quality control of the Illumina MiSeq Platform output data was conducted using Trimmomatic software (Bolger et al., 2014), which filtered bases with a reads tail mass value of < 20 to ensure high quality reads for subsequent analysis. The full-length ITS2 rDNA fragments were obtained using the consolidated PEAR data (Zhang et al., 2014), read quality was trimmed, and chimeras checked using MOTHUR. CUTADPAT was used to trim the reverse and forward primers sequences. The precise methods used can be found in Ziegler et al. (2017).

We found that the previous ITS2 databases contained some replicate sequences, so we collected several published databases (Franklin et al., 2012; Arif et al., 2014; Tong et al., 2017) and uploaded them to the CD-HIT Suite Website^2^, set sequence identity cut-off as 100%, and established a non-duplicate ITS2 database (Supplementary Data Sheet 1).

In order to cope with the challenge of using the ITS2 gene as a multicopy marker, we used sequence-based ITS2 analysis and OTU analysis to evaluate Symbiodiniaceae diversity and community composition in the SCS. This methodology provide a comprehensive description of molecular diversity and biogeograhpical patterns of Symbiodiniaceae in the SCS, because sequence-based ITS2 analysis can directly assess the association between coral and symbionts, and OTU-based analysis is able to estimate Symbiodiniaceae molecular richness and diversity requiring no formal description of ITS2 symbiont types (Ziegler et al., 2017).

For sequence-based ITS2 analysis, the quality-filtered reads were aligned to the ITS2 database using BLASTn and the parameters setting following the pipeline detailed in Tong et al. (2017) (Supplementary Table 2). In addition, in order to allow comparison with the results of DGGE from previous studies and to avoid IGV interference, we determined the number of different ITS2 sequences that were present at a minimum cut-off of > 5% in at least one of the 178 samples (Ziegler et al., 2017).

For OTUs analysis, qualified ITS2 sequences were subsampled to 1000 reads per sample using MOTHUR, and sequences with a retention length of > 90% were clustered into an OTU based on a similarity of 97% (Arif et al., 2014; Thomas et al., 2014; Ziegler et al., 2017). The most abundant OTU sequence was selected as the representative sequence to be aligned to the ITS2 database using BLASTn, and non-Symbiodiniaceae OTUs were removed (Arif et al., 2014). Finally, OTU data were used for a statistical assessment of difference in Symbiodiniaceae diversity and community composition (Supplementary Table 4).

Venn and PERMANOVA were analyzed using GNU R software. CANOCO 5.0 was applied to the CCA to establish the relationships between zooxanthellae ITS2 subclade and environmental factors (Šmilauer and Lepš, 2014). The phylogenetic tree was constructed based on the Kimura two-parameter model with uniform rates between sites using Bayesian inference in MrBayes.

## Results

### Symbiodiniaceae Diversity in the SCS

For ITS2 sequence-based analysis, 7,761,607 sequences were identified that belong to Symbiodiniaceae, after quality control. *Symbiodinium*, *Breviolum*, *Cladocopium*, *Durusdinium*, *Fugacium*, *Gerakladium*, Clade H, and Clade I were detected in the sequences. However, in order to reduce the impact of intragenomic diversity on the results, only ITS2 variants that were present with at least 5% abundance in at least one sample were considered. Consequently, we were able to identify *Cladocopium*, *Durusdinium*, and *Gerakladium*, and these were therefore retained for further analysis. At the subdivision level, 31 dominant Symbiodiniaceae types (number of sequences: 7,420,489, covering more than 95%) were identified from three genera, namely *Cladocopium* (*n* = 26), *Durusdinium* (*n* = 4), and *Gerakladium* (*n* = 1).

For OTU-based analyses, the subsampled data set contained 178 coral samples throughout the SCS, representing Symbiodiniaceae ITS2 sequences which clustered into 58 OTUs at a 97% similarity. These OTUs included five Symbiodiniaceae genera, namely *Symbiodinium* = 1 OTU, *Breviolum* = 1 OTU, *Cladocopium* = 46 OTUs, *Durusdinium* = 5 OTUs, and *Gerakladium* = 5 OTUs.

**Figure 2:**
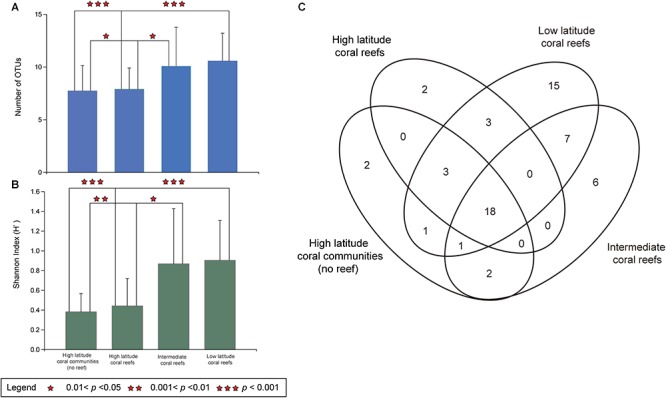
Symbiodiniaceae OTU richness and diversity in hosts of five coral species in the South China Sea. **(A)** The number of OTUs of Symbiodiniaceae in five coral species (*Porites lutea*, *Favia palauensis*, *Plesiastrea versipora*, *Motipora efflorescens*, and *Acropora formosa*) from distinct latitude regions. The error bars are mean SD. **(B)** The Shannon (H’) diversity index values of Symbiodiniaceae in five coral species from different latitude regions. The error bars are mean SD. **(C)** Venn diagram showing the number of Symbiodiniaceae OTUs were identified in high latitude coral communities (no reef), high latitude coral reefs, intermediate coral reefs and low latitude coral reefs in the South China Sea as well as number of OTUs that were shared between them.

In addition, the LR contained 47 Symbiodiniaceae OTUs and had the highest regional Symbiodiniaceae OTU diversity (*n* = 15). The IR contained 33 Symbiodiniaceae OTUs and six regional OTUs (Figure 2C). In comparison, HC and HR contained 24 and 27 OTUs, respectively, and two regional OTUs (Figure 2C). Interestingly, 18 OTUs were found to be present in all regions (17 OTU in *Cladocopium* and 1 OTU in *Durusdinium*). This account for 31.0% of all OTUs detected and was higher than the number of regional OTUs in any of the regions studied (Figure 2C).

Furthermore, there was a significant spatial difference in the OTU richness of Symbiodiniaceae, which was higher in the LR than in the HC (Kruskal-Wallis, *p* = 0.0001 < 0.001) or the HR (Kruskal-Wallis, *p* = 0.0001 < 0.001). Equally, the Symbiodiniaceae OTU richness was also higher in the IR than in the HC (Kruskal-Wallis, *p* = 0.021 < 0.05) or the HR (Kruskal-Wallis, *p* = 0.035 < 0.05). However, there was no significant difference in OTU richness between the LR and the HR (Kruskal-Wallis, *p* = 0.076 > 0.05) nor between the HC and HR (Kruskal-Wallis, *p* = 0.6408 > 0.05; Figure 2A). In addition, the results of Kruskal-Wallis test indicated that the variation in the Shannon (H’) index values and OTU richness were consistent (Figure 2B). Consequently, the diversity and richness of Symbiodiniaceae OTUs in the IR and LR was determined to be higher than in the HC or the HR.

### Community Composition of Symbiodiniaceae in the SCS

**Figure 3:**
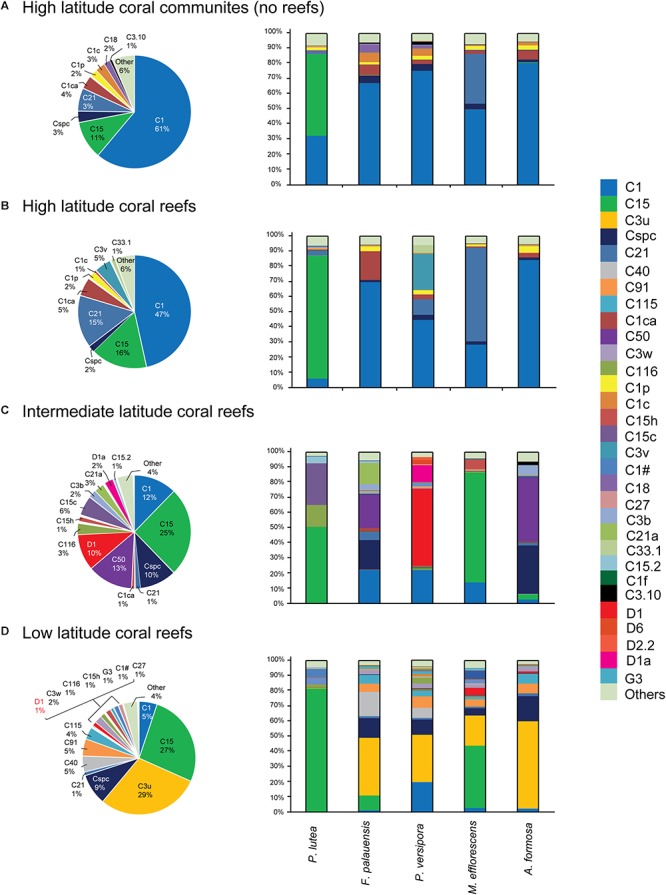
Five coral species associated with Symbiodiniaceae ITS2 type-based community composition from the SCS. **(A)** High latitude coral communities (no reef). **(B)** High latitude coral reefs. **(C)** Intermediate latitude coral reefs. **(D)** Low latitude coral reefs. Those sequences were contained that represented relative abundance > 5% in at least one sample in this community analysis.

The Symbiodiniaceae community composition was determined across sampling sites by analysis of OTUs in each region that had more than 5% relative abundance in at least one ITS2 sequence sample (Figure 3). The results of this analysis show that all of the regions studied are dominated by ITS2 sequences from *Cladocopium*, *Durusdinium*, and *Gerakladium*. At the subdivision level, the community composition of five corals species had undergone distinct changes, with the exception of the stable symbiotic relationship between *Porites lutea* and C15 that was observed in all four latitude regions (Figure 3). In HC and HR, the Symbiodiniaceae composition was dominated by C1 and C15, but C15 only made a large contribution to *Porites lutea* (Figure 3A,B).

In comparison, the composition of communities in warmer IR were largely dominated by C50, Cspc, C15, C1, and D1. It is worth noting that the relative abundance of C15, C50, Cspc, and D1 increased in IR communities, while the contribution of C1 decreased moving from the high latitude region (HC and HR) to the IR. C15 dominated the community composition of *Porites lutea* and *M. efflorescens*, while C50 and Cspc made large contributions to the community composition of *F. palauensis* and *A. formosa*. In addition, the relative abundance of heat-tolerant D1 also increased in *Plesiastrea versipora*, compared with HC and HR (Figure 3C).

C3u dominated communities in the LR, which had high contributions from *F. palauensis*, *Plesiastrea versipora*, *M. efflorescens*, and *A. formosa*. The contribution of C1 to Symbiodiniaceae community composition was lower in the LR, compared with the IR (Figure 3D). Generally, going from the HC and HR through the IR to the LR, the symbiont community shift is represented by a decreasing contribution from C1, and an increasing contribution of C15. There is also a large contribution from C50, Cspc, and D1 in IR and C3u in the LR. Accordingly, our results showed that there were differences between the community compositions of Symbiodiniaceae dominated types at different latitude regions.

**Figure 4:**
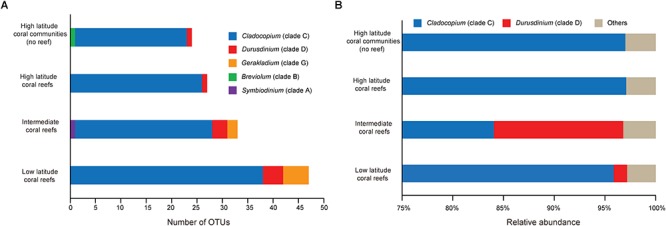
The OTU-based Symbiodiniaceae genera composition and relative abundance over the five species coral samples in each latitude regions. **(A)** Stackplot showed Symbiodiniaceae OTU-based genera richness in each regions. **(B)** Symbiodiniaceae OTU-based community structures at genus level.

Based on the results of the OTU analysis, OTUs belonging to *Cladocopium* (HC:22, HR:26, IR:27, and LR:38) and *Durusdinium* (HC:1, HR:1, IR:3, and LR:4) were identified in all latitude regions, while OTUs assigned to *Gerakladium* were found in the IR (*n* = 2) and LR (*n* = 5). In addition, one OTU assigned to *Symbiodinium* was only detected in the IR, and one OTU belonging to *Breviolum* was only found in the HC (Figure 4A). Moreover, the relative abundance of more than 1% Symbiodiniaceae OTUs indicated that the community composition of HC and HR only contained OTUs belonging to *Cladocopium*. In contrast, the IR and LR not only contained OTUs belonging to *Cladocopium*, but also to *Durusdinium*. In addition, the relative abundance of OTUs belonging to *Breviolum*, *Symbiodinium*, and *Gerakladium* was no more than 1% (Figure 4B). A PERMANOVA analysis also showed that there were significant differences in the OTU community composition of regions from different latitudes (*F* = 14.75, *R*^2^ = 0.20, *p* = 0.001 < 0.01).

### The Relationships Between Environmental Factors and the Symbiodiniaceae

**Figure 5:**
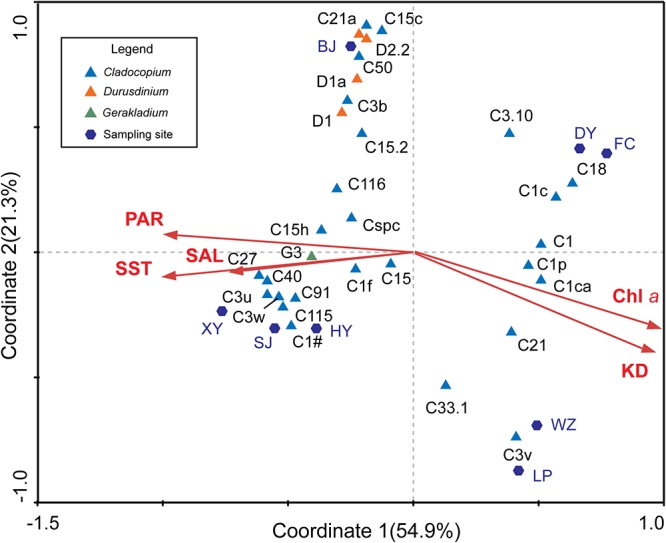
Relationships between Symbiodiniaceae types and environmental factors. The CCA indicated the relationship among the relative abundance of 31 dominant Symbiodiniaceae types, environmental and sampling areas. The first axis (Coordinate 1) explains 54.9% of the total variation and the second axis (Coordinate 2) explains 21.3% of the total variation.

According to the CCA, the SST, PAR, SAL, Chl *a*, and KD were all potential impact factors that affected Symbiodiniaceae types and community composition in the SCS (Figure 5). The impact factors of Chl *a* and KD were negatively correlated with SST, PAR, and SAL in the *X*-axis, combined with sampling sites, which roughly indicated the change of environmental factors from the subtropics to tropics. The night Symbiodiniaceae types (C1, C3.10, C1c, C18, C1p, C1ca, C21, C33.1, and C3v) were positively correlated with Chl *a* and KD, but negatively correlated with SST, PAR, and SAL. The night types mostly occurred in DY, FC, WZ, and LP. In contrast, the other 22 Symbiodiniaceae types were negatively correlated with Chl *a* and KD, and were positively correlated with SST, PAR, and SAL, which mostly occurred in BJ, HY, SJ, and XY. This result was consistent with the community composition determined by ITS2 sequence analysis.

In addition, we obtained the ITS2 sequence of the dominant Symbiodiniaceae type in the SCS from a database (Supplementary Data Sheet 1) and created a phylogenetic tree based on Bayesian inference. Combined with the CCA analysis results, the members of *Durusdinium* and *Gerakladium* were all positively correlated with Chl *a* and KD, and negatively correlated with SST, PAR, and SAL in the SCS. Interestingly, the phylogenetic and environmental characteristics of *Cladocopium* were distinct from other Symbiodiniaceae genera. Most of the members of the Symbiodiniaceae type have a close phylogenetic relationship with C1, were positively correlated with Chl *a* and KD, and negatively correlated with SST, PAR, and SAL in the SCS. By comparison, most members of the Symbiodiniaceae type have a close phylogenetic relationship with C3 and C15, which were positively correlated with Chl *a* and KD, and negatively correlated with SST, PAR, and SAL in the SCS. However, C1f, C21, and C3.10 did not show any clear correlation between the phylogenetic relationships and environmental factors in the SCS.

## Discussion

### Biogeographical Pattern of Symbiodiniaceae Types in the SCS

Although we detected OTUs that belong to *Symbiodinium*, *Breviolum*, *Cladocopium*, *Durusdinium*, and *Gerakladium*, the relative abundance of ITS2 sequences (relative abundance < 5%, Figure 3 and Supplementary Table 2) and OTUs (relative abundance < 1%, Figure 4B) that belong to *Symbiodinium* or *Breviolum* are very low. Therefore, we suggest that the ITS2 variants from *Symbiodinium* and *Breviolum* are presently at low abundance in corals, and that they may be transient, rather than stable, symbionts (Lee et al., 2016). Alternatively, they may have been incorrectly detected due to interference from IGV (Thornhill et al., 2007).

Based on the results of ITS2 sequences and OTU analysis, we conservatively suggest that only three genera of Symbiodiniaceae, *Cladocopium*, *Durusdinium*, and *Gerakladium*, have symbiotic relationships with coral in the SCS. This is the first time that the presence of *Gerakladium* in the SCS had been confirmed. Moreover, we found that, in addition to *Porites lutea*, the other four coral species played a relatively minor role in determining the dominant Symbiodiniaceae types. Symbiodiniaceae communities in the SCS are generally dominated by two or three Symbiodiniaceae types, such as C3u and Cspc, which are widely distributed in LR corals (Figure 3D). Although there are differences in the Symbiodiniaceae community composition of *F. palauensis*, *Plesiastrea versipora*, *M. efflorescens*, and *A. formosa* in IR, they all have a certain proportion of C1 (Figure 3C). Consequently, the different environmental factors between the four latitude coral habitats could potentially explain the differences in Symbiodiniaceae community composition.

*Cladocopium* and *Durusdinium* are the main Symbiodiniaceae genera found in stony corals throughout the SCS. This result is consistent with previous studies based on traditional detection methods (such as PCR-DGGE and RFLP, Chen et al., 2005; Zhou and Huang, 2011). Based on NGS, we found that there are abundant Symbiodiniaceae ITS2 variants from *Cladocopium* and *Durusdinium* in the SCS. This contradicts the view that corals have a low level diversity of Symbiodiniaceae ITS2 types in this region (Chen et al., 2005; Zhou and Huang, 2011; Liu et al., 2012; Ng and Ang, 2016). Our study was the first to confirm the presence of *Gerakladium* in the SCS, and to determine that it is mainly found in the IR and LR (Figure 4). The relative abundance of G3 correlated positively with SST, PAR, and SAL, and correlated negatively with Chl *a* and KD (Figure 5). *Gerakladium* is an ecologically rare genus (LaJeunesse et al., 2018) and occurs predominantly in the Pacific Ocean (Pochon et al., 2001, 2006; Oppen et al., 2005; Schoenberg and Loh, 2005). In addition, *Gerakladium* has been detected at a low abundance as background symbionts in stony coral colonies (LaJeunesse et al., 2018). However, while our level of knowledge of *Gerakladium* is comparatively limited, research into its geographical distribution has shown that this genus prefers high SST and SAL survival environments, such as the Arabian Sea (Ziegler et al., 2017). Thus, the biogeographical pattern of *Gerakladium* also supports the viewpoint that the geographical distribution and community composition of Symbiodiniaceae may be mainly determined by environmental factors.

### The Latitudinal Flexibility of Symbiodiniaceae Richness and Diversity

The richness and diversity of Symbiodiniaceae types in the IR and the LR were higher than those in the HC and HR, based on an OTU analysis (Figure 2). Biogeographical and ecological studies have shown that the center of Symbiodiniaceae diversity overlaps with the “Coral Triangle” (LaJeunesse et al., 2012), which is close to the IR and LR and far away from the HC and HR. The physiological evidence suggests that Symbiodiniaceae swim just 3–10 m over the course of one day (Fitt and Trench, 1983), and have a lifespan of approximately seven days in their natural environment (Nitschke, 2015). They are most likely dependent on sea currents for dispersal (Wirshing et al., 2013; Thornhill et al., 2017). Therefore, due to geographical distance limitations, only a small number of Symbiodiniaceae taxa may have spread to the HC and HR, which may also be one of the reasons why the richness and diversity of Symbiodiniaceae in the HC and HR are lower than in the IR or LR.

In addition, the environmental stresses present in the HC and HR, which are caused by seasonal SST fluctuations and high nutrient concentrations, may also cause the observed limitations in Symbiodiniaceae diversity and distribution. It has been found that Symbiodiniaceae diversity in the Persian/Arabian Gulf was limited by extreme environmental conditions, with high SST and elevated salinity (D’Angelo et al., 2015; Ziegler et al., 2017). Furthermore, the abundant inorganic nitrogen content can lead to Symbiodiniaceae using nutrients for their own growth and reproduction (Dubinsky and Jokiel, 1994), meaning that they compete with coral for the carbon dioxide required for calcification (Szmant, 2002). As a result, high nutrient concentrations will reduce the stability of the symbiotic relationships between Symbiodiniaceae and corals (Marubini and Davies, 1996; Szmant, 2002). The symbiotic relationship between coral and Symbiodiniaceae is relatively stable (Thornhill et al., 2006; Hume et al., 2015), which is probably due to co-evolution over a long period of time. However, high nutrient concentrations can lead to this relationship becoming unstable, which may lead to the deaths of both the coral and the Symbiodiniaceae symbionts. Therefore, higher environmental resistance in HC and HR may also result in the elimination of Symbiodiniaceae types that have poor adaptability or unstable symbiotic relationships with coral.

It is worth noting that the number of OTUs (*n* = 18, 31.0%) that were distributed across all four regions is higher than the number of regionally unique OTUs (HC:2, HR:2, IR:6, LR:15, Figure 2C). In addition, only four unique OTUs occurred in the HC and HR. Therefore, corals contained many different Symbiodiniaceae OTUs in the HC, HR, IR, and LR, while the corals of the IR and LR have more regional Symbiodiniaceae OTUs (Figure 2C). This result supports the view that Symbiodiniaceae in high latitude coral habitats may originate from low latitude regions, or the “Coral Triangle” in the SCS. Although there has not yet been research conducted on the genetic connectivity of Symbiodiniaceae in the SCS, there have been some studies on corals (Su, 2017; Huang et al., 2018). For example, Huang et al. (2018) identified that the gene flow of *Porites lutea* was universally asymmetrical northward in the SCS, which possibly reflects the northward migration of the coral (Huang et al., 2018). Other studies have found that coral range expansion was accompanied by a reduction in the diversity of Symbiodiniaceae genotypes (Serrano et al., 2013; Grupstra et al., 2017). Furthermore, many Symbiodiniaceae OTUs were distributed across four latitude regions (Figure 2C), which suggests that many kinds of Symbiodiniaceae types may have a relative wide range of environmental adaptations than previously considered. The Symbiodiniaceae type that dominate symbiont community composition in different latitude coral habitats in the SCS may depend on their distinct competitive characteristics. Pettay and LaJeunesse (2013) found that the genotypes of *Durusdinium glynii* populations in the subtropical Gulf of California are significantly differentiated from population in tropical eastern Pacific, and they suggested that this may be due to strong adaptive genotypes that are selected because of their resistance to extreme environments.

### Symbiodiniaceae Community Composition Was Affected by Multiple Environmental Factors in the SCS

In our study, the Symbiodiniaceae community shifted from C1 and C15 dominance in the HC and the HR, via decreasing proportions of C1 and increasing proportions of C15, C50, Cspc, and D1 in the IR, to a Symbiodiniaceae community dominated by C3u and C15, and to a lesser extent by C1, in the LR (Figure 3). Interestingly, while both C1 and C15 belong to *Cladocopium*, their contribution to the community differed dramatically with the change in latitude; C1 was abundant at latitudes where C15 was scarce, and C15 was abundant in latitudes where C1 was scarce (Figure 3). Although the relative abundance of C15 in the community composition of different regions is largely determined by *Porites lutea*, the relative abundance of C15 is obviously higher in *F. palauensis* and *M. efflorescens* in the IR and LR, despite the widely established symbiotic relationship with C1 in the HC and HR. Such environments are found in high latitude marginal coral communities in Okinawa, Japan and Jeju Island, Korea, and C1 was the primary dominant symbiont in many host Symbiodiniaceae communities (Reimer et al., 2006; Palmas et al., 2015). By contrast, C15 exhibited particularly strong thermal tolerance (Pochon et al., 2004), which allows it to thrive in high heat-stress coral habitats, such as the Andaman Sea (LaJeunesse et al., 2010). Accordingly, SST shaped the Symbiodiniaceae community composition; the increased relative abundance of Durusdinium in the IR and LR also supports this viewpoint. A recent study by Tong et al. (2017) on *Galaxea fascicularis* and *Montipora* spp. in the SCS showed that SST was one of the key impact factors for the Symbiodiniaceae community composition.

It is noteworthy that D1, D2.2, D1a, and D6, which belong to *Durusdinium*, were not dominant in the LR, and occurred more frequently in the IR in association with *Plesiastrea versipora* (Figure 3D, 4). The result of the ITS2 sequence-based analysis showed that C3u was dominant in the community composition of the LR, while the contribution of D1 was only 1% (Figure 3D). The relative abundance of *Durusdinium* OTUs in the IR was higher than that in LR (Figure 4B). As a result, the community contribution of *Durusdinium* did not always increase as latitude decreased in the SCS. In addition, C3u may be a symbiont with a potentially strong heat tolerance, and it has been found in offshore coral habitats in the Andaman Sea that are known for exhibiting high water temperatures (LaJeunesse et al., 2010). Furthermore, the relative abundance of C3u was higher than that of *Durusdinium* in offshore, while *Durusdinium* is more commonly distributed in inshore in areas of the Andaman Sea with higher turbidities and nutrition concentrations (LaJeunesse et al., 2010). Consequently, because coral species have little effect on the composition of the Symbiodiniaceae type present, the weaker competitiveness of *Durusdinium* compared with C3u may be because of the lower turbidity and nutrition concentrations in the IR. Studies have suggested that nutrition concentration and turbidity have the potential to impact the Symbiodiniaceae community composition (Sawall et al., 2014; Ng and Ang, 2016; Tong et al., 2017; Gong et al., 2018). For example, Gong et al. (2018) found that, in addition to SST, nutrient inflow can affect coral–algal symbiotic associations. Equally, Ng and Ang (2016) discovered that the low richness and diversity of Symbiodiniaceae in Hong Kong coral habitats may be due to high turbidity. In contrast, *Durusdinium* is more readily able to adapt to environments with high SST, high turbidity, and tidal cycles (LaJeunesse et al., 2010, 2018; Pettay et al., 2015). Moreover, many symbionts that belong to *Cladocopium* also have high heat tolerance, such as *Cladocopium thermophilum* (formerly C3-Gulf), C41, and C39, which all occur in regions of the Persian Gulf with extremely high SST and elevated salinities (D’Angelo et al., 2015; Hume et al., 2016; Ziegler et al., 2017). Within the SCS, the relative abundance of numerous symbionts belonging to *Cladocopium* positively correlated with SST, PAR, and SAL, and these symbionts were mostly found in the LR (Figure 5). These symbionts seem to show strong adaptability to high SST seawater environments, and high competitiveness in such environments. Although the Symbiodiniaceae community is mainly shaped by SST, the impact of other environmental factors, especially nutrients and turbidity, cannot be neglected.

### Distant Ancestors May Affect Relationships Between the Phylogenetic Relationships and Environmental Adaptability of Symbiodiniaceae in the SCS

**Figure 6:**
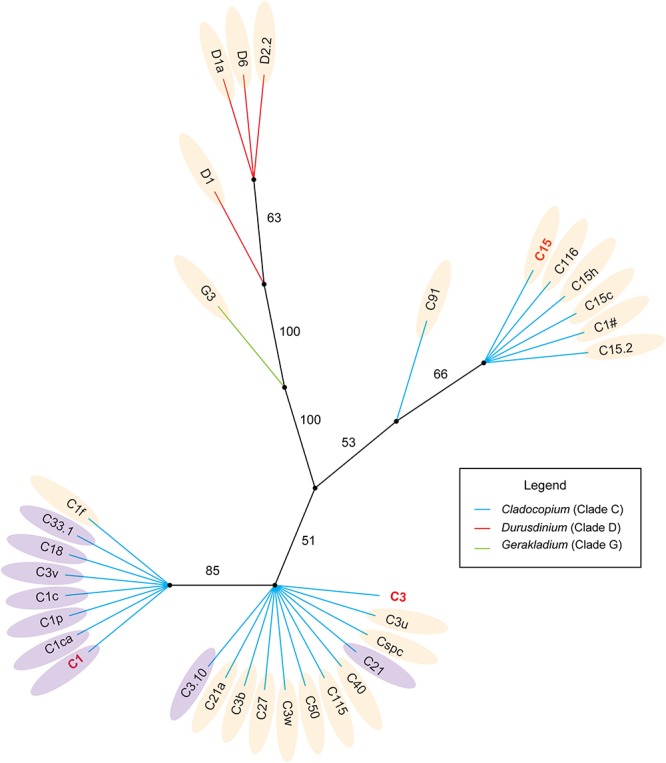
Phylogenetic analysis of the dominant Symbiodiniaceae ITS2 types in the SCS based on Bayesian inference. The light orange areas mean those subclades were positively correlated with SST, SAL, PAR and negative correlated with Chl *a* and KD; The lilac areas mean those subclades were negatively correlated with SST, SAL, PAR and positively correlated with Chl *a* and KD.

Some members of the C1 and C3 types may be the ancestors of *Cladocopium* (LaJeunesse, 2005; Thornhill et al., 2014). The CCA and the phylogenetic tree both provide evidence that the members of *Cladocopium* have a close phylogenetic relationship with C1, the relative abundance of which was negatively correlated with SST, PAR, SAL, and was positively correlated with Chl *a* and KD (Figure 5, 6). In contrast, the *Cladocopium* symbionts have closer phylogenetic relationships with C3, the relative abundance trend of which showed the opposite pattern to the C1 group (Figure 5, 6). Some Symbiodiniaceae types of *Cladocopium* with close phylogenetic relationships may be similar in terms of their environmental adaptability, and distant ancestors might affect the environmental adaptability of members of the clades present in the SCS. C15 as a putative younger clade that derived from C3 (LaJeunesse, 2005), and the relative abundance of C15 was positively correlated with SST, PAR, and SAL (Figure 5, 6). In addition, *Cladocopium* has high OTU richness (Figure 4A), but because *Cladocopium* was the most species rich, ecologically abundant, and functionally diverse genus within the Symbiodiniaceae (Reimer et al., 2006; LaJeunesse et al., 2018), the ecological functions of members this genus are difficult to predict and evaluate. Our study found, through a combination of phylogenetic and CCA analyses, that it may be possible to speculate regarding the environmental adaptability of Symbiodiniaceae types in the SCS. This method has been applied to the ecological study of Symbiodiniaceae (LaJeunesse, 2005; LaJeunesse et al., 2010; Gong et al., 2018). For example, Tong et al. (2017) used the dominant Symbiodiniaceae type (which has a relative abundance of more than 10%) ITS2 sequence to construct a phylogenetic tree. This revealed that the phylogenetic relationships of the Symbiodiniaceae types were associated with geographical distances. LaJeunesse et al. (2010) also used this method to determine that 5% of the *Cladocopium* species characterized were unique to the Indian Ocean, and that many of these were regionally endemic.

However, associations between phylogenetic relationships and environmental adaptability were not stable, for example C1f within the C1 group, or C3.10 and C21 within the C3 group (Figure 6). In addition, the presence of intragenomic rDNA variation can potentially confound estimates of symbiont diversity and may have interfered with our identification of Symbiodiniaceae types in ITS2 marker-based analyses (Thornhill et al., 2007; Stat et al., 2011). Moreover, the correlation between phylogenetic relationships and environmental adaptability may also be due to sequence types that represent IGV from within a single Symbiodiniaceae species (Thornhill et al., 2007). Therefore, Symbiodiniaceae species with the same environmental adaptability within a group may not be as rich as expected. Future ecological and evolutionary studies of Symbiodiniaceae may need to utilize analyses of markers that provide detailed genetic resolution, and the use of psbA^ncr^ is essential (LaJeunesse and Thornhill, 2011; Reimer et al., 2017). However, phylogenetic analysis based on the ITS marker can provide a rough framework for evolutionary research based on high-resolution markers, to ensure they are categorized at the appropriate taxonomic level of Symbiodiniaceae (Thornhill et al., 2014; Pettay et al., 2015).

## Author Contributions

KY and BC designed the research. JL, WH, GW, HS, XH, ZQ, ZP, and YW contributed the materials. BC, WL, and YL performed the research. BC and JL analyzed the data and drawn all pictures. BC and KY wrote the manuscript. All authors reviewed the manuscript.

## Conflict of Interest Statement

The authors declare that the research was conducted in the absence of any commercial or financial relationships that could be construed as a potential conflict of interest.

## Footnotes

1 https://giovanni.gsfc.nasa.gov/giovanni/

2 http://weizhongli-lab.org/cdhit_suite/cgi-bin/index.cgi?cmd=cd-hit-est
