# Supplementary material for: Latitudinal Variation in the Molecular Diversity and Community Composition of Symbiodiniaceae in Coral From the South China Sea
Source: Front Microbiol. 2019 Jun 18;10:1278. doi: 10.3389/fmicb.2019.01278 (PMC6591375; doi:10.3389/fmicb.2019.01278)
Supplement: Supplementary file 3 [file Table_3.DOCX]

Supplementary Material

# **Latitudinal variation in the diversity and community composition of Symbiodiniaceae ITS2 types and its adaptive potential to respond to climate change in the South China Sea**

Biao Chen, Kefu Yu*, Jiayuan Liang, Wen Huang, Guanghua Wang, Hongfei Su, Xueyong Huang, Zhenjun Qin, Ziliang Pan,Wenwen Luo, Yanqiu Luo, Yinghui Wang

*** Correspondence:** Dr. Kefu Yu : kefuyu@scsio.ac.cn

# Supplementary Data

See Appendix S1,S2, and S4

# Supplementary Figures and Tables

## Supplementary Figures

## **
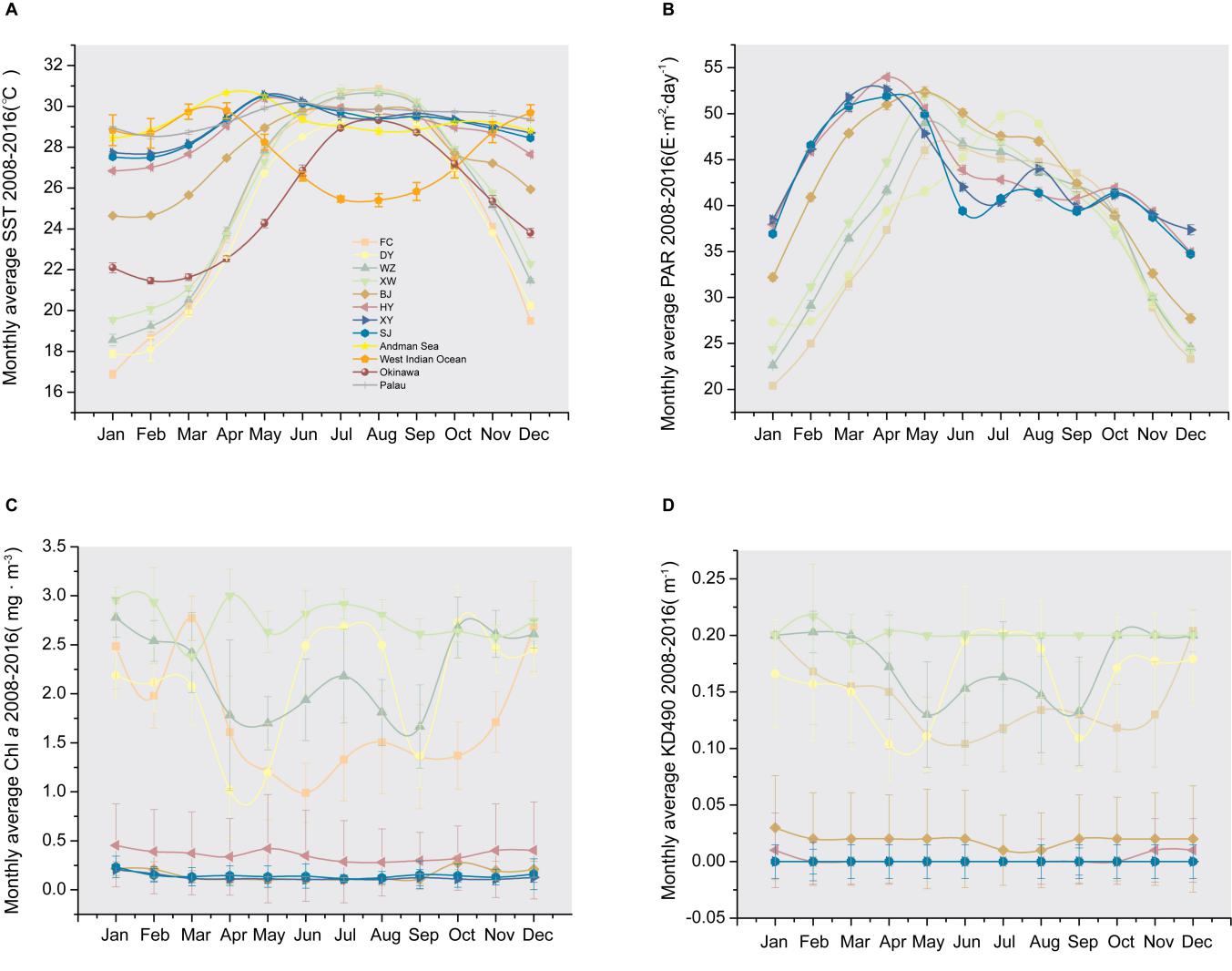
**

**Figure S1 Monthly average of SST, Chl a, PAR and KD in the South China Sea.** The data was collected from NASA Giovanni satellite (https://giovanni.gsfc.nasa.gov/giovanni/)


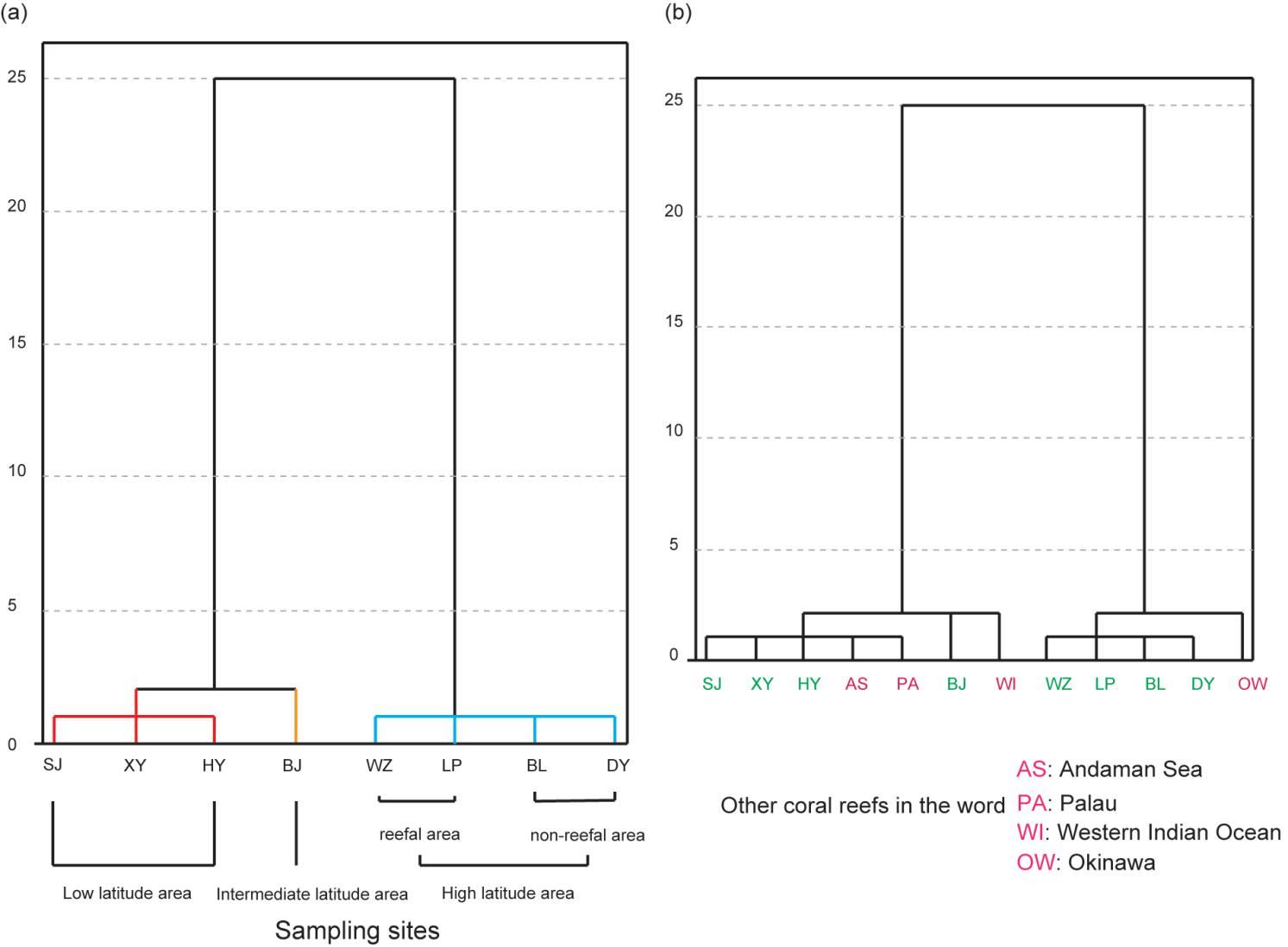


**Figure S2.** Ward dendrogram using squared Euclidean distance clustering of sampling site SST metric.
